# Osteomyelitis of the hyoid bones in two calves

**DOI:** 10.1186/s13028-015-0147-6

**Published:** 2015-09-22

**Authors:** Karl Nuss, Alexandra J. Malbon, Ueli Braun, Simone Ringer, Evelyne Muggli, Patrick Kircher, Florian Willmitzer

**Affiliations:** Department of Farm Animals, Vetsuisse Faculty University of Zürich, Winterthurerstrasse 260, 8057 Zurich, Switzerland; Institute of Veterinary Pathology, Vetsuisse Faculty University of Zürich, Winterthurerstrasse 260, 8057 Zurich, Switzerland; Section of Anaesthesiology, Equine Department, Vetsuisse Faculty University of Zürich, Winterthurerstrasse 260, 8057 Zurich, Switzerland; Department of Small Animals, Clinic of Veterinary Diagnostic Imaging, Vetsuisse Faculty University of Zürich, Winterthurerstrasse 260, 8057 Zurich, Switzerland

**Keywords:** Calf, Anorexia, Oral inflammation, Hyoid bone, Osteomyelitis

## Abstract

Two calves were referred because of ptyalism and difficulty opening the mouth (Calf 1) and for elective umbilical hernia surgery under inhalation anaesthesia (Calf 2). Additional clinical signs were increased breath sounds and swelling in the region of the mandibular angle in Calf 1. Ultrasonography and endoscopy revealed oral inflammation and abscessation in the area of the base of the tongue in both calves. Infection of the hyoid apparatus was suspected based on ultrasonographic findings and confirmed by means of computed tomography. In Calf 1, there was no response to treatment with systemic antibiotics, nonsteroidal anti-inflammatory drugs and local lavage, and Calf 2 was not treated. Both calves were euthanized because of a poor prognosis and the diagnoses confirmed during postmortem examination. In Calf 1, the abscess was associated with complete destruction of the left epihyoid bone and partial destruction of the left stylohyoid and ceratohyoid bones. In Calf 2, the abscess was located at the distal end of the right stylohyoid bone near the epihyoid bone. Stomatitis or laryngeal and pharyngeal abscessation caused by sharp feed particles are common in cattle and infection of the hyoid apparatus should be included in the differential diagnosis.

## Background

Mechanical dysphagia in calves is predominantly caused by diffuse inflammatory processes or abscesses that result from foreign bodies or injuries from rough feed particles; the tongue and larynx are most commonly affected [1]. Injuries of the oropharynx and larynx are associated with considerable inflammation of the upper neck region, swelling, and abnormal respiratory noises [2, 3]. Clinical, ultrasonographic, radiographic, or endoscopic examinations may identify the nature of the swelling [1, 4, 5] and in some cases allow targeted treatment. To our knowledge, involvement of the stylohyoid bone in infectious inflammatory conditions of the oral cavity in cattle has not been described but osteopathies or fractures of the stylohyoid bone are common in horses [6–10]. The hyoid apparatus plays a crucial role in the swallowing process by moving the food bolus over the larynx. The hyoid body, which is seated between the larynx and the tongue, consists of the basihyoid and the paired thyrohyoid and ceratohyoid bones. Two branches of the hyoid apparatus function as suspension for the body and consist, from distal to proximal, of the paired epihyoid, stylohyoid and tympanohyoid bones [11]. Diseases of the stylohyoid bones in adult horses are referred to as temporohyoid osteoarthropathy because of their typical localization and nature. This condition may involve the middle ear, the temporohyoid joint, the stylohyoid bone and the petrous portion of the temporal bone. Possible aetiologies discussed in the literature include extension of infection from otitis media or interna, guttural pouch infection, strangles, trauma and osteoarthritis [6–10]. Clinical signs include chronic weight loss, nasal discharge when the guttural pouch is affected, paralysis of the tongue and locked jaw, and the prognosis is unfavourable. Temporohyoid osteoarthropathy is accompanied by bony proliferation and fusion of the temporohyoid joint. The restriction of mobility is believed to lead to stress fractures of the petrous temporal and/or stylohyoid bones.

## Case presentation

Calf 1 was a 6-month-old Brown Swiss heifer calf with a 3-week history of anorexia, ptyalism and swelling of the throat region. Examination of the oral cavity by the referring veterinarian revealed an injury in the region of the left mandibular angle. The calf was treated with an analgesic (meloxicam) and an antibiotic (spiramycin), but did not respond to treatment and was referred to the Department of Farm Animals, Vetsuisse Faculty. Calf 2 was a 3-month-old Holstein–Friesian heifer calf referred for elective umbilical hernia surgery. No other abnormalities were reported and the calf appeared healthy on clinical examination except for increased breath sounds.

**Fig. 1:**
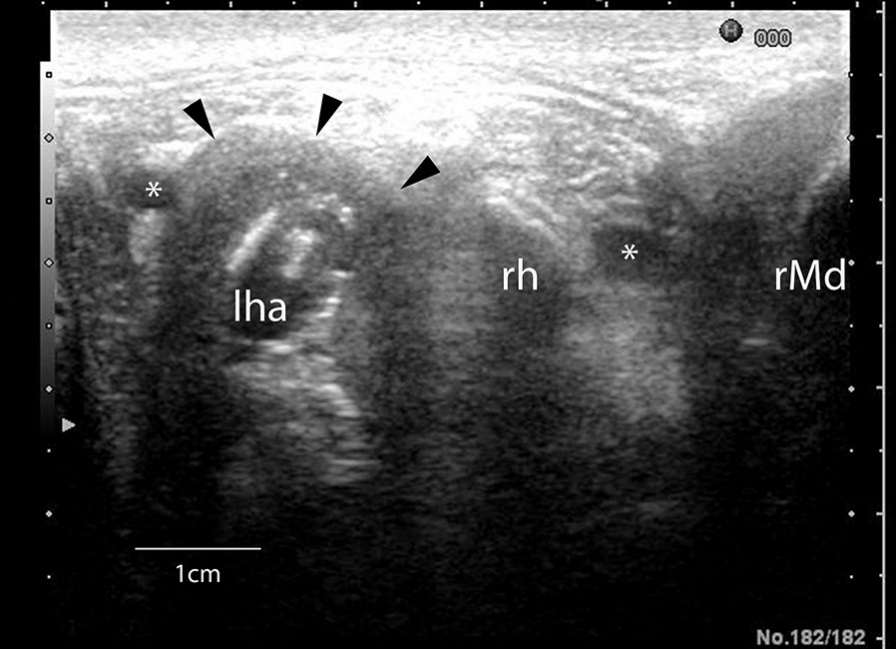
Ultrasonogram of the throat area in a 6-month-old Brown Swiss heifer calf (Calf 1). Ultrasonogram of the throat area at the level of the base of the tongue showing an abscess associated with the left hyoid bones. Ventral is at the *top*. *rMd* right mandible, *rh* right hyoid, abscess in the area of the left hyoid delineated by *black arrow heads*, *asterisk* lingual arteries

**Fig. 2:**
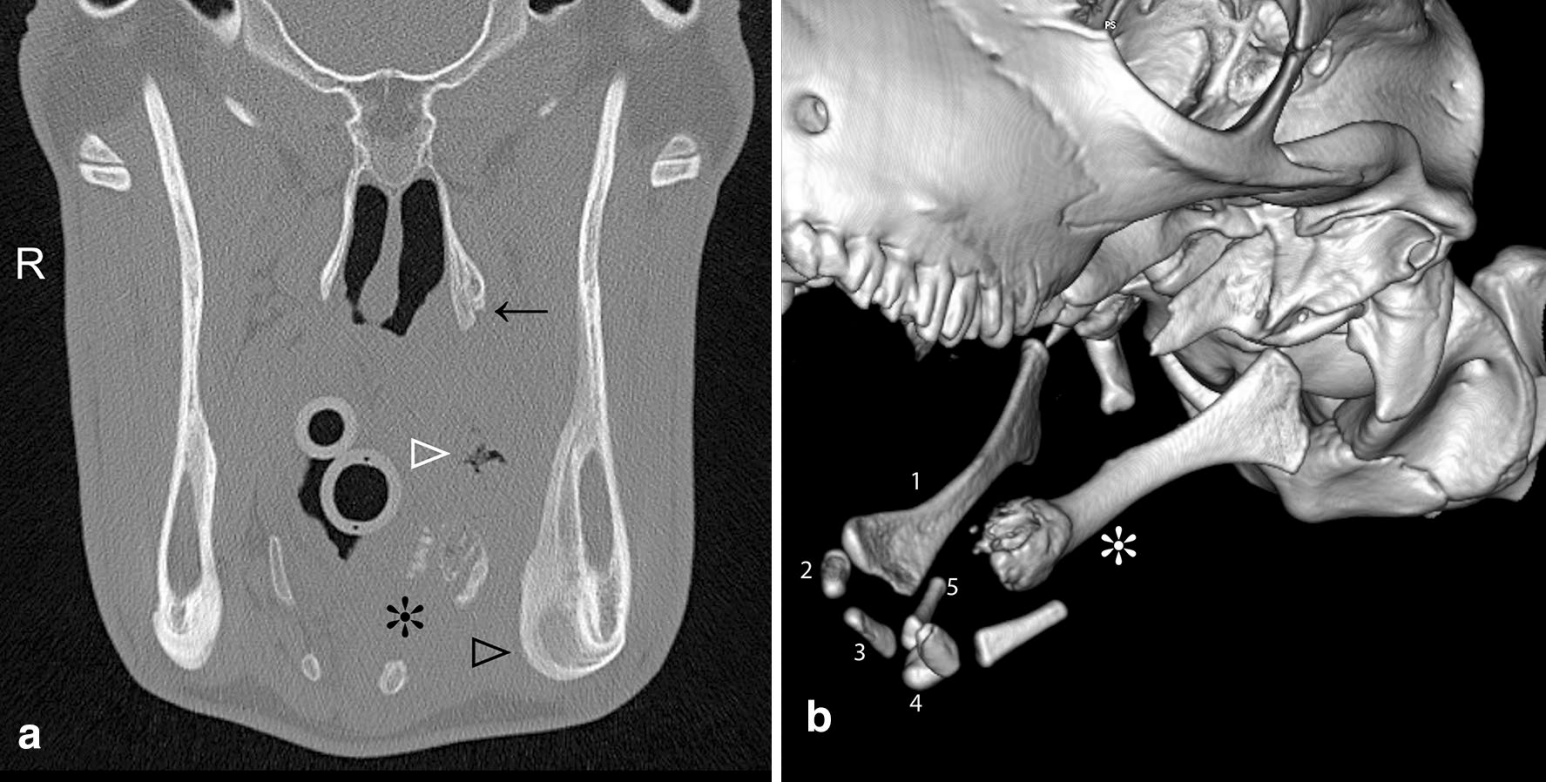
CT transverse image and 3D reconstruction of a 6-month-old Brown Swiss heifer calf with stylohyoid osteomyelitis (Calf 1). **a** Transverse CT image at the level of the stylohyoid bone. The distal left stylohyoid bone is partly distended and osteolytic (*asterisk*), there is an irregular gas inclusion within the abscess (*white arrow head*), moderate smooth and solid periosteal new bone formation ventromedially at the left mandible (*black arrow head*) and mild periosteal new bone formation at the pterygoid bone (*black arrow*). **b** 3D reconstruction of the hyoid apparatus viewed from the left. The distal left stylohyoid bone is partially expanded and osteolytic (*asterisk*) and the left epihyoid bone is missing. Bones of the right hyoid apparatus are numbered, *1* stylohyoid, *2* epihyoid, *3* ceratohyoid, *4* basihyoid, *5* thyrohyoid; not identified: tympanohyoid

**Fig. 3:**
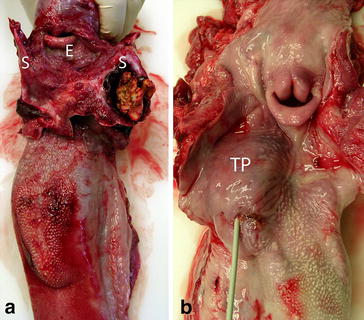
Postmortem views of oral abscesses associated with the hyoid apparatus in two calves. **a** The abscess in Calf 1 contained inspissated necrotic and purulent material and involved the epihyoid bone and the distal part of the left stylohyoid bone. The entire pharyngeal area was inflamed (*S* Stylohyoid, *E* Epiglottis). **b** In Calf 2, the abscess was located on the right at the base of the tongue in the region of the tonsillar fossa (probe) and palatine tonsil (TP) and involved the distal end of the stylohyoid bone

Clinical examination revealed that Calf 1 was depressed, had a poor body condition and a starry hair coat. There was ptyalism, bilateral mucoid nasal discharge and increased breath sounds in addition to a non-painful oedematous swelling in the region of the left mandibular angle and inability to fully open the mouth. No lesions were seen on lateral radiographic views. Ultrasonography with a 10–5 MHz linear probe showed an abscess between the left mandible and the base of the tongue. The calf was sedated [xylazine, 0.1 mg/kg bodyweight (bw) intramuscularly (IM)] for endoscopy, which revealed a fistulating abscess in the region of the left mandibular angle that extended from the base of the tongue to the third molar of the upper jaw. Pus and necrotic tissue were removed through the fistula using a curette. Crystalline sodium penicillin (22,000 IU/kg bw, four times a day) was administered intravenously (IV) for 2 days followed by the same dose of procaine penicillin administered daily IM for another 10 days. Flunixin (2.2 mg/kg bw/day IV) was given postoperatively for 3 days. The abscess was flushed every other day for 8 days with the calf sedated. The demeanour and appetite of the calf improved transiently, and there was mild improvement in its ability to open the mouth but the abscess did not regress clinically or ultrasonographically. Ultrasonographic re-examination showed that a part of the hyoid apparatus was osteolytic and involved in the abscess (Fig. 1). The calf was placed under general anaesthesia and computed tomography (CT) was carried out using a 40 slice CT unit. Transverse images and three-dimensional reconstruction revealed extensive destruction of the left hyoid apparatus (Fig. 2). Based on all the findings, a diagnosis of hyoid osteomyelitis was made and the calf was euthanized while still under anaesthesia because of a poor prognosis. Postmortem examination showed a firm fistulated swelling on the left side of the caudal aspect of the tongue, which partially occluded the larynx (Fig. 3a). Irregular tracts extended toward the ventral aspect of the tongue medial to the stylohyoid bone, forming a cavity with a smooth dark red to black lining and a thick creamy to friable content. The tracts ran through salivary and tonsillar tissues but tissue destruction was severe and the origin of the tracts obscured by inflammation. The left mandible was approximately three times thicker than normal and its size increased gradually from rostral to caudal. The maximum thickness of the left stylohyoid bone was 24 mm compared with the normal right stylohyoid bone, which was 4 mm, at its junction with the epihyoid bone. Bacteriological culture of material from the oral lesions yielded moderate numbers of *Trueperella pyogenes* and small numbers of *Fusobacterium necrophorum* and *Clostridium sporogenes*.

**Fig. 4:**
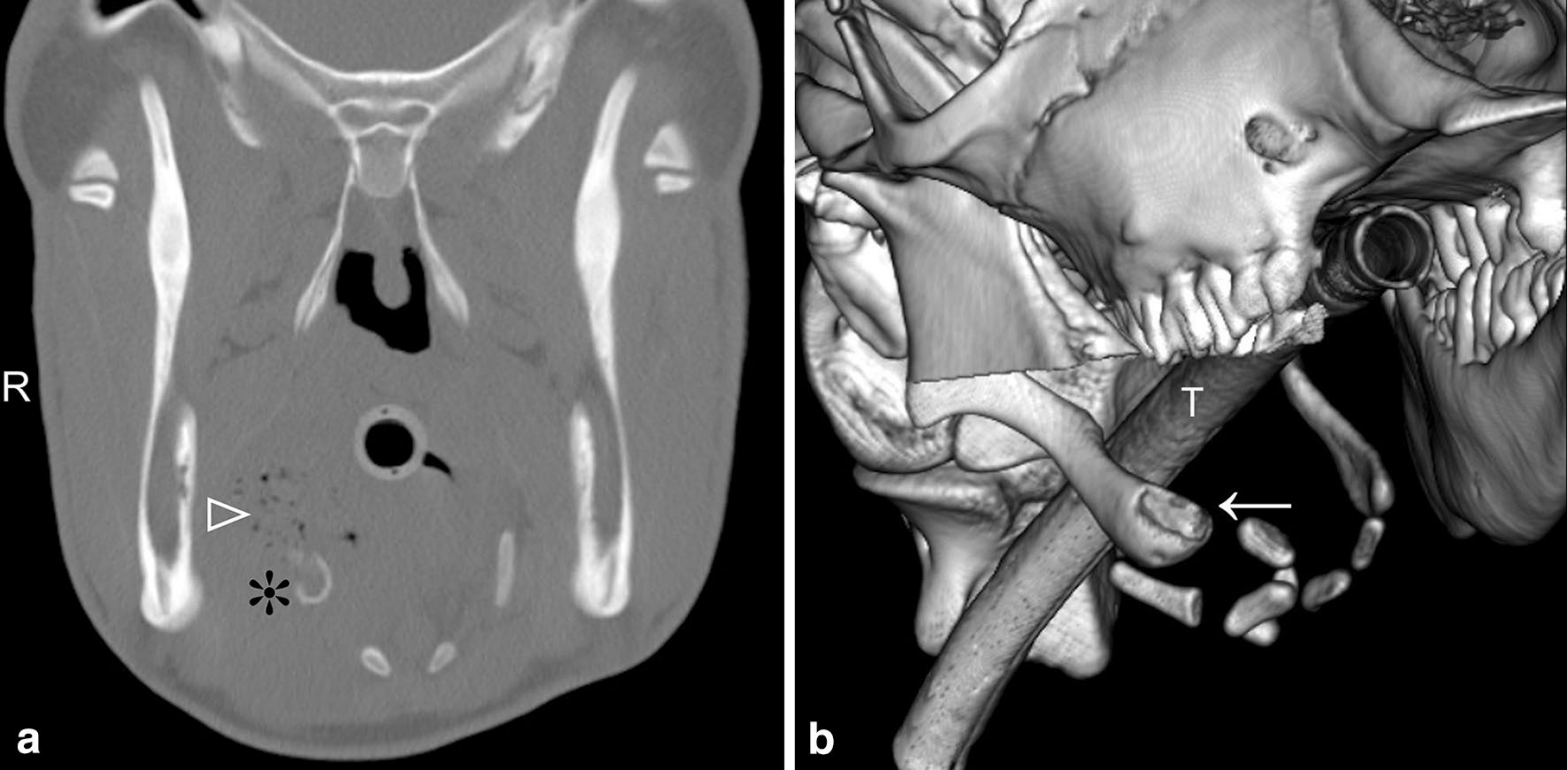
CT transverse image and 3D reconstruction showing stylohyoid osteomyelitis in a 3-month-old Holstein–Friesian heifer calf (Calf 2). **a** Transverse CT image at the level of the stylohyoid bone showing inflammation with multiple small gas inclusions (*white arrow head*) in the region of the base of the tongue on the right side and osteolytic changes of the right stylohyoid bone (*asterisk*). **b** 3D reconstruction of the hyoid apparatus viewed from the right. There is marked remodelling and early osteolysis of the distal part of the right stylohyoid bone (*white arrow*)

In Calf 2, a swelling was detected between the lower right third molar and the base of the tongue during endotracheal intubation for general anaesthesia. Inhalation anaesthesia was carried out but instead of umbilical surgery, the oral cavity was examined using a variety of imaging modalities. Endoscopy showed a 5 × 5 cm fistulated dark red swelling on the right side of the base of the tongue. Ultrasonographic examination showed an abscess at the base of the tongue under the tongue musculature involving the hyoid apparatus. CT examination with the calf in dorsal recumbency showed bony proliferation and osteolysis of the distal part of the right stylohyoid bone near the articulation with the epihyoid bone, and a large abscess with gaseous content in this region (Fig. 4). This calf was diagnosed with hyoid osteomyelitis and euthanized because of a poor prognosis. Postmortem examination showed a 5 cm swelling dorsolateral to the base of the tongue on the right side caudal to the *torus linguae* with a fistula at the rostral aspect (Fig. 3b). The fistula and the swelling were connected to the hyoid apparatus at the articulation between the right stylohyoid and epihyoid bones. The fistulous tract led to an irregular cavity with a 2 cm wall of firm granulation tissue and multiple masses consisting of homogeneous, yellow, inspissated pus at the centre. Most of these agglomerates could be easily detached from the abscess cavity except for the ones that were connected to the necrotic cartilage of the articulation between the stylohyoid and epihyoid bones. Bacteriological culture of material from the oral lesions yielded moderate numbers of *Streptococcus mutans*, *Bacteroides* sp., and *F. necrophorum* and small numbers of *Lactobacillus salivarius*.

## Conclusions

Among the clinical signs observed in Calf 1, difficulty opening the mouth was of particular interest because this was a characteristic finding in horses with infected stylohyoid bone fracture [7]. It is likely that the bony proliferations of the mandibular and stylohyoid bones and the inflammation contributed to the inability of the calf to open its mouth completely. Infection of the hyoid apparatus in both calves was probably caused by feed particles that were forced caudally during swallowing and perforated the pharyngeal mucosa. The organisms isolated are oral commensals and colonise lesions following traumatic damage of the overlying mucosa. *Trueperella pyogenes* in particular is a common opportunistic pathogen in cattle, and *F. necrophorum* and *Bacteroides* spp. are recognised as important primary pathogens, causing oral and laryngeal necrobacillosis [12].

The distal portion of the stylohyoid bones and the epihyoid bones are the structures of the hyoid apparatus [11] closest to the oro-pharyngeal mucosa and thus the most likely bones to be involved in an oro-pharyngeal infection. Another possibility is the spread of an infection from the palatine tonsil since the inflammation was located in this area in Calf 2. In the calves reported here, it is conceivable that the infection started at the cartilaginous articulations and then spread to the epihyoid and stylohyoid bones. Osteolytic changes of the ceratohyoid bone have also been described in horses although usually the articulation between the stylohyoid and temporal bones is affected [7, 10], occasionally accompanied by middle ear infection [9]. However, there was no evidence of otitis externa in the calves reported here and CT scans did not show inner or middle ear abnormalities. Surgical treatment of osteomyelitis of the hyoid bones was not considered feasible because of the location of the lesion and the destructive changes seen in CT image reconstruction. Furthermore, functional impairment of the hyoid apparatus would be expected after any form of treatment and therefore both calves were euthanized. One report of successful surgical treatment of a stylohyoid bone fracture associated with an abscess in a horse has been published but a description of the surgical technique was not provided [6].

In summary, in calves with swellings in the throat and mandibular area, particularly in association with difficulty opening the mouth, disorders of the hyoid apparatus should be included as a differential. The diagnosis can be confirmed by means of ultrasonography and CT. Although the latter is not an absolute prerequisite for a definitive diagnosis, it allows precise characterization of the disease process and the involvement of bony structures.
